# The Effects of Mild Cognitive Impairment on Fall Severity in Older Adults

**DOI:** 10.1093/geroni/igab046.2617

**Published:** 2021-12-17

**Authors:** Megan Jones, Sally Paulson, Joshua Gills, Anthony Campitelli, Jordan Glenn, Erica Madero, Jennifer Myers, Michelle Gray

**Affiliations:** 1 University of Arkansas at Fayetteville, Fayetteville, Arkansas, United States; 2 University of Arkansas at Fayetteville, Cincinnati, Ohio, United States; 3 Neurotrack Technologies, Redwood City, California, United States

## Abstract

Falls affect more than 30% of older adults and are one of the leading causes of injury, hospitalization, and mortality in this populations. Mild cognitive impairment (MCI) is one of the risk factors for falls in older adults. The purpose of this study is to determine if older adults with MCI have increased fall severity than older adults without MCI. Participants (n: 81: age: 79 ± 6) completed a Montreal Cognitive Assessment (MoCA) and a Hopkins Falls Grading Scale, a tool used to grade the severity of falls on a scale of 1-4 (1 = loss of balance without fall; 4 = fall requiring hospital admission). Participants were categorized as having MCI (score <26: N: 44: age: 81 ± 6.4) or non-MCI (score ≥26: n: 37: age: 77 ± 6). Groups were analyzed using a one-way ANOVA in SPSS to compare the severity of falls within the previous 12 months. There were no differences between groups for fall grade 1 (p =.22) or fall grade 2 (p =.45). There was a significant difference between groups for fall grade 3 (p =.04) and fall grade 4 (p =.05) with the MCI group having more of these falls compared to the non-MCI group. Older adults with MCI had a higher number of falls requiring medical attention than older adults without MCI. Although falls are a risk in all older adults, those with MCI may be at higher risk of more injurious falls than older adults without MCI.

